# Effects of hyperthyroidism and diabetes mellitus on spermatogenesis in peri- and post-pubertal mice

**DOI:** 10.3389/fendo.2023.1191571

**Published:** 2023-08-15

**Authors:** Hanhao Shi, Nazar Ali Korejo, Asghar Ali Kamboh, Rashid Ali Korejo, Fangxiong Shi

**Affiliations:** ^1^ College of Animal Science and Technology, Nanjing Agricultural University, Nanjing, China; ^2^ College of Animal Science and Technology, Anhui Agricultural University, Hefei, China; ^3^ Faculty of Animal Husbandry and Veterinary Sciences, Sindh Agriculture University Tandojam, Hyderabad, Pakistan; ^4^ Department of Animal Nutrition, Faculty of Animal Production and Technology, Shaheed Benazir Bhutto University of Veterinary and Animal Sciences, Sakrand, Pakistan

**Keywords:** diabetes, hyperthyroidism, testis, epididymis, spermatogenesis, mouse

## Abstract

**Introduction:**

Diabetes and thyroid dysfunction often co-exist. One autoimmune disorder always invites another and it has been reported that such co-morbid ailments always become detrimental to the health of the patients.

**Materials and methods:**

In our previous work, we elucidated the interactions of diabetes and hypothyroidism on testicular development and spermatogenesis. However, the present study illuminates the interface between diabetes and hyperthyroidism, where 16 ICR pregnant primiparous mice were used and subsequently 48 male pups were randomly selected (n=12/group) and separated into 4 groups: control (C), diabetic (D), diabetic + hyperthyroidism (DH) and hyperthyroidism (H).

**Results:**

Computerized sperm analyses showed significant reductions in count by 20% and increases of 15% in D and H animals, respectively, vs. controls. However, rapid progressive sperm motility was significantly lower only in D (30%) compared with C mice. Our histomorphometric investigation depicted damaging effects on testicular and epididymal tissues; the stroma adjacent to the seminiferous tubules of the D mice revealed edematous fluid and unstructured material. However, in the epididymis, germ cell diminution contraction of tubules, compacted principal and clear cells, lipid vacuolization, atypical cellular connections, exfoliated epithelial cells, and round spermatids were conspicuous in DH mice.

**Discussion:**

Collectively, our experiment was undertaken to ultimately better recognize male reproductive disorders in diabetic-hyperthyroid patients.

## 1 Introduction

Organ-specific autoimmune disorders are developing in certain individuals, and many families have become victims of such syndromes (1). Persistent hyperglycemia can affect thyroid gland activity, one of the important endocrine systems in animals (2). The two most frequent endocrine ailments encountered in clinical practices are the thyroid diseases and diabetes mellitus (3). The high occurrence of autoimmunity of thyroid (AIT) and thyroid malfunctioning have been reported in type-1 diabetic patients (4). The strong association between diabetes and thyroid diseases stimulated the American Diabetes Association (ADA) to recommend that people with diabetes be tested sporadically for thyroid malfunctioning (5). Many diabetic individuals have also been extensively reported to have reproductive alterations (6), the relationship between the two endocrine disorders has been described in various groups during the past two decades (7–9). It is reported that deiodinases and thyroid receptors are presented in the testis, and thousands of novel genes in testes that are regulated by T3 (1, 3). It certainly affects the glucose metabolism which is critical in the testis function. Testes regulate the carbohydrate metabolism in turn. For example, testosterone which mainly secreted from the testis in male animals are involved in carbohydrate metabolism via direct effects on skeletal muscle, liver, adipose tissue, and immune cells and indirectly through changes in body fat mass and distribution (10).

Thyroid malfunctioning has been found to be predominant among diabetic populations when compared with non-diabetics (11). In our previous study, we examined the concomitant effects of diabetes mellitus and hypothyroidism on spermatogenesis (12). However, the regarding synchronous effects of diabetes mellitus and hyperthyroidism on spermatogenesis are scarce.

The consequences of the concomitant metabolic disturbances on the different systems of the body have been debated only retrospectively based upon clinical case records in humans, but the data were seen as deficient in the context of research trials for the investigation of such syndromes and their connections with reproductive fitness. This investigation attempts to mimic such complications where multiple glands are involved by producing experimental DM concurrently with hyperthyroidism through injections of STZ and levothyroxine, respectively. The consequences of these co-morbid metabolic indices were examined in the morphology of testis and epididymis, along with a quantitative evaluation of endocrine hormones and spermatogenesis in the germinal epithelia of the testis.

## 2 Materials and methods

### 2.1 Ethics statement

The investigational procedures involving mice were directed in agreement with the Guide for the Care and Use of Laboratory Animals organized by the Institutional Animal Care and Use Committee of Nanjing Agricultural University, China. Authorization regarding the use of laboratory animals in our university was certificated by No. SYXK (Su) 2017–0007, and the ethics approval number of this project is NAU2015018 from our university ethics committee.

### 2.2 Experimental animals and treatments

Sixteen primiparous ICR-strain female mice, pregnant for approximately 15 days were purchased from the Qinglongshan Laboratory Animal Company (Nanjing, China). These animals were maintained at a controlled temperature (21–22°C), light cycle (12h light vs 12h dark), and humidity (65%–70%). Tags were made for all mice and mice were offered standard balanced mouse pellets as feed with drinking water *ad libitum*. Following parturition, 48 male pups were randomly selected (n=12/group) and distributed into 4 groups: C, D, DH, and H. We used cold citrate buffer (pH 4.4) to dissolve STZ for immediate use prior to injection. Diabetes was induced in pups of groups D and DH by 3 intra-peritoneal injections of STZ at a dose of 40 mg/kg on postnatal days 3, 4 and 8 (13). The induction of diabetes in pups was proposed on postnatal day 3 because most of the testicular cell population started to develop at about the same day in mice (14). The second reason was to avoid maternal aggression leading to pup cannibalism. The post-delivery females of groups DH and H were made hyperthyroid by injecting 0.3 mg/kg body weight of levothyroxine on a daily basis (15). After weaning (24 d), half of the pups were sampled and observed, and the other half continued to receive the same hyperthyroid treatment until 56 days of age. The total duration of treatment to the experimental animals was 8 weeks.

### 2.3 Collection of samples

Regarding the animals (n=6/group) that were sampled at 24 d (weaning), following measurement of body weights, the animals were anesthetized with halothane for collection of blood samples and then euthanized by cervical dislocation. The weight of both testes and epididymides were recorded and the left testis and epididymis were fixed in 4% paraformaldehyde for histologic analysis. Blood serum was isolated by centrifugation of the samples at 4000 g for 10 min., and the sera were stored at −80°C until further use.

Spermatozoa of 56-day-old mice were collected according to our laboratory methods (16). We carefully collected each cauda from all mice and following washing with normal saline at 37°C, they were transferred to 1.5-ml Eppendorf tubes containing 500 µl of artificial human tubular fluid (HTF, 37°C) medium (17).

### 2.4 Biochemical assays

The random blood glucose levels of every mouse were assayed during blood sampling with a Sannuo rapid blood glucose meter (Sinocare Inc., Changsha, China). The maximal limit of the device is 27.8 mmol/L, so the values above that we denoted as 28 mmol/L. We determined serum concentrations of hormones by using commercial radioimmunoassay (RIA) kits (North Institute of Biotechnology, Beijing, China) at the General Hospital of the Nanjing Military Command, Nanjing, China. The sensitivity for insulin-like growth factor 1 (IGF-1), testosterone (T), free thyroxine (fT4), and free triiodothyronine (fT3) were <5 ng/ml, 0.02 ng/ml, 1 fmol/ml, and 0.5 fmol/ml, respectively. The intra- and inter-assay coefficients of variation for all hormones (IGF1, T, fT4, and fT3) were <10% and <15%, respectively.

### 2.5 Sperm quality assessment

#### 2.5.1 Sample preparation

After 8 weeks of treatment, cauda epididymides were isolated, cleaned once in normal saline, separated from fat and adjoining tissues, and transferred to 500 µl of sperm suspense on medium (HTF). The cauda epididymides were incubated at 37°C in 5% CO2/95% air for 5 minutes, minced 5-7 times inside the tube, and left for 15 min under the same conditions to allow the release of sperm into the medium.

#### 2.5.2 Sperm count

A Neubauer chambered slide was used for counting sperm at a 1:20 dilution (18),. We then measured the average sperm density in millions/millimeter by counting them in the 4 large corners and the center squares.

#### 2.5.3 Sperm motility

Briefly, for the sperm motility assessment, we prepared a 10-µl sample to perform computer-assisted sperm analysis (CASA), and for every single evaluation, we analyzed 30 frames in 0.5 sec and made 6 measurements, with a total of at least 2000 spermatozoa for each animal (16).

### 2.6 Histomorphometric analyses

The fixed-tissue samples were dehydrated through a graded series of alcohol, cleared in xylene, and embedded in paraffin. The 5-μm thick sections were cut and stained with hematoxylin and eosin (HE). Three independent observers, unaware of the slide identity, observed histo-morphologic changes under a light microscope (Nikon, Tokyo, Japan). Epithelial cells, gametes, and interstitial spaces were examined, noting extent of epithelial thickening, and diameter and size of the lumen in micrometers. The morphometric measurements were done by microscopic calibration under 400× magnification in accordance with systematic technique of microscopic investigation (12).

### 2.7 Statistical analysis

Graph Pad Prism (Version 5.0) software was used for computations. Values are presented as means ± standard error of the mean (SEM). The variances of groups were calculated with 1-way ANOVA, followed by Tukey’s post-hoc test and 2-way ANOVA by considering Bonferroni post-hoc tests to compare the means of the replicates. A P-value of <0.05 was considered significant.

## 3 Results

### 3.1 Body, testes and epididymal weights

**Figure 1:**
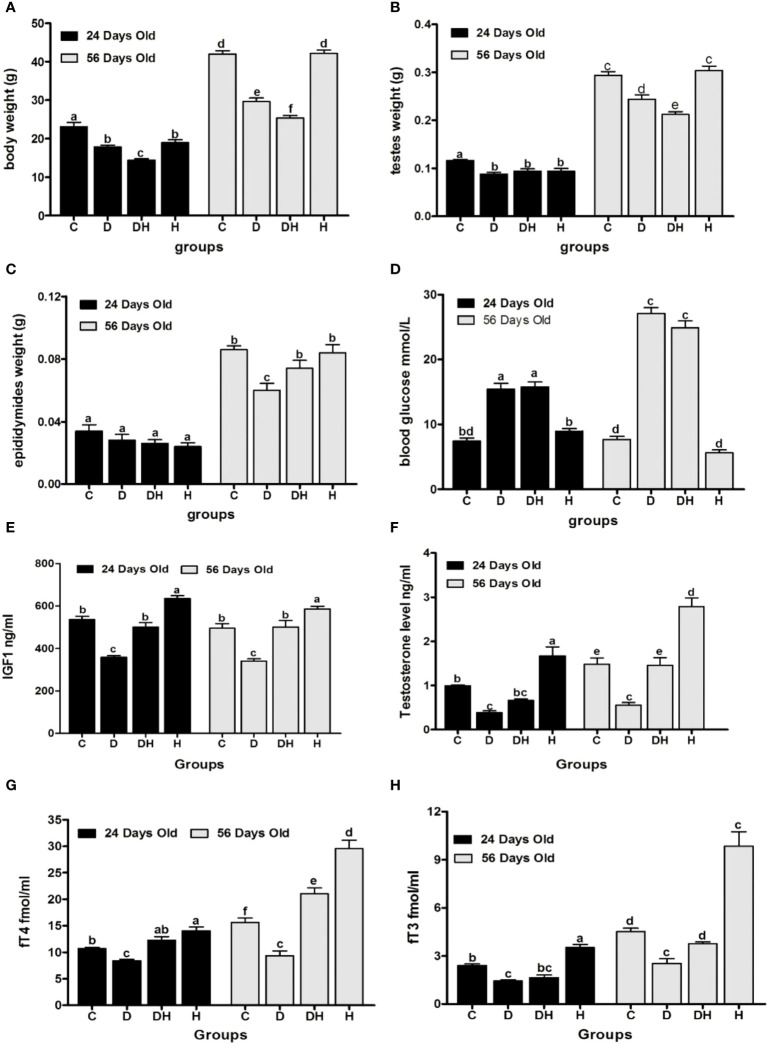
Effects of STZ-induced diabetes and hyperthyroidism on body, testes, and epididymal weights, with random blood glucose levels and serum concentration of different hormones of pre- (24 d) and post-pubertal (56 d) mice. Part Labels shows; **(A)** body weight, **(B)** testes weight, **(C)** epididymides weight, **(D)** random blood glucose level, **(E)** IGF1, **(F)** testosterone level, **(G)** free thyroxine level, **(H)** free triiodothyronine level. Each bar represents a mean (*n*=6) with SEM-vertical line on the top. The data were analyzed across groups by 2-way ANOVA by considering Bonferroni post- hoc tests to assess differences within and between groups of the 2 age classes (i.e. 24 days vs. 56 days). Different labels on Figure bars indicate significant (*P*<0.05) discrepancies among groups.

**Figure 2:**
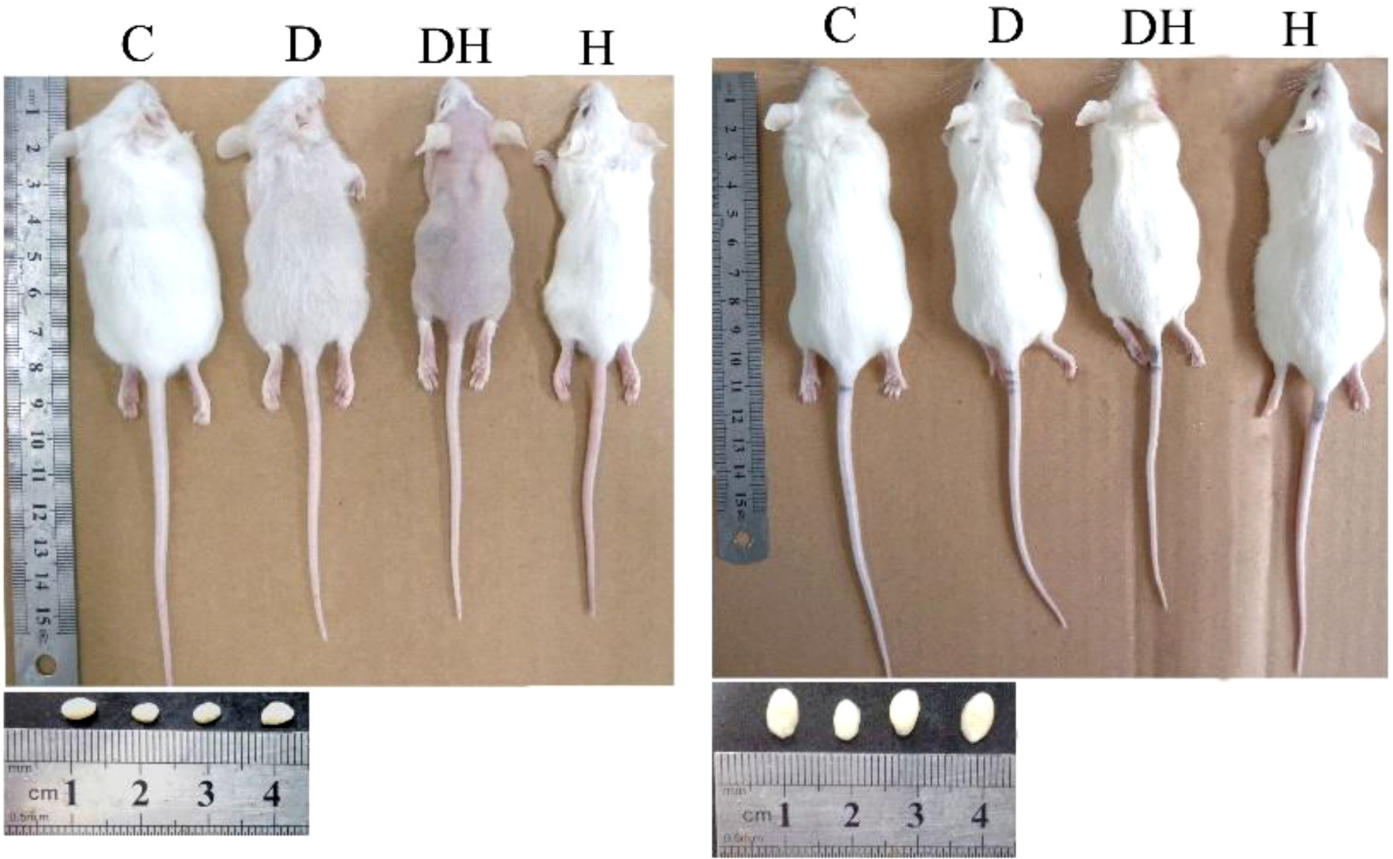
Photographs of mice and correlating testes at the different age levels; i.e., 24 (left panel) and 56 days of age (right panel). Each mouse in this photograph was randomly selected from each group and photographs were captured using a Cannon (Power Shot SX700 HS) digital camera.

Body weights of experimental animals were recorded before sacrifice. The pre-pubertal mice showed decreased body weights following the induction of diabetes and hyperthyroidism; however, it was profoundly diminished in the DH (by 32%) mice in comparison to controls (**Figures 1A**, **2**). The 80% of pre-pubertal mice in the DH and D groups exhibited no and fewer hairs on their body, respectively. However, this type of disorder disappeared, when they entered the age of puberty (**Figure 2**). When these mice entered the age of puberty at day 56, the mice of DH were found consequently suffering from lower weights (40%), followed by D (29%) compared with control animals. However, the animals receiving levothyroxine (0.3 mg/kg body weight) daily reset their body weights to normal levels with no statistical differences in comparison to control animals.

### 3.2 Blood glucose and hormone levels

Following the induction of diabetes with STZ, >95% animals were observed to be in the state of polydypsia, polyphagia and polyuria until the end of the trial. At the time of sacrifice, the random blood glucose levels (**Figure 1D**) of immature animals were significantly elevated in the D (108%) and DH groups (112%), while at 56 d of age, these values were markedly higher in the D (254%) and DH (225%) groups in comparison to the control group. However, the control and hyperthyroid mice showed no statistical differences during the short- and long-term study periods.

Serum levels of IGF-1 (**Figure 1E**) were remarkably decreased in STZ-diabetic mice, however, it was found to be significantly elevated in hyperthyroid animals in both study periods. Hyperthyroidism consistently augmented IGF-1 levels of DH mice to the same level as controls. The growth rate of animals remained at a peak level just before and after puberty, and this, we believe, is why IGF-1 values showed no overall statistical significance between pre- and post-pubertal periods.

Serum testosterone levels (**Figure 1F**) in pre-pubertal mice were significantly decreased in D (52%) and elevated in H groups (68%) compared to controls. However, the DH animals showed distinctly attenuated values for testosterone that were comparable only to H mice. Similarly, post-pubertal mice exhibited minimal and maximal values of testosterone in D (62%) and H (88%) groups, respectively in comparison to controls. However, at this stage the DH group showed higher values than D, but were still not significantly different from control animals.

The pre-pubertal mice exhibited markedly reduced fT4 levels (**Figure 1G**) in diabetic (21%) mice, and increased in the H group (31%) compared to controls. However, fT4 was diminished in D (16%) and elevated in H groups (22%) followed by DH (14%) animals compared with controls in the longer period. Collectively, the lowest values for fT4 were seen in D animals at both times.

During the short-study period, blood fT3 levels (**Figure 1H**) were significantly decreased in the D group (40%), however, these were increased by 41% in the H group compared with control mice. Similarly, in adult animals the values were noticeably lower in D (44%) and higher in H groups (118%) compared with controls, although STZ-induced diabetes circumvented the rise in fT3 values in the DH relative to C animals at both times.

Our results suggest that effects of short-period hyperthyroidism on blood fT4 and fT3 values in neonatal mice can be ameliorated by breastfeeding compared with treatment in adults.

### 3.3 Morphometric measurements of testicular and epididymal tissues at different ages

We performed the histomorphometric analysis of the seminiferous and epididymal tubules of pre- and post-pubertal mice (**Table 1**) through microscopic calibrations. The diameter of the seminiferous tubules of pre-pubertal mice was found to be markedly decreased in all treated animals compared with controls, while during the post-pubertal period, we found it to be profoundly reduced only in the DH group compared with all other experimental animal groups. However, in comparison to controls, the lumen diameter of these tubes increased significantly in D (56%) and DH (35%) groups in the short-term and DH (36%), D (31%), and H (17%) in the long-term study. We observed shrinkage of germinal epithelium in all treated mice during both study periods compared with controls; however, a marked decrement was only noted in the DH group (30%), which was chronically exposed to these co-morbid diseases. The diameter of the seminiferous tubules along with their lumen diameter and epithelial height rose significantly with age between the 2 study periods.

**Table 1:** Morphometric measurements of different parts of seminiferous and epididymal tubules in micro meters (µm) in STZ-diabetic and hyperthyroid mice through microscopic calibrations.

day	item	Control	Diabetic	Diabetic +Hyper	Hyper
**Pre-pubertal (24d)**	St. diameter	147.2 ± 2.4^a^	129.2 ± 2.3^b^	133.9 ± 2.3^b^	122.9 ± 1.4^b^
	St. Lumen diameter	34.5 ± 2.6^b^	53.7 ± 2.4^a^	46.1 ± 2.9^a^	34.0 ± 2.5^b^
	St. Epithelial height	54.8 ± 1.2^a^	42.1 ± 1.4^d^	47.5 ± 1.1^bc^	48.6 ± 1.7^b^
	Caput diameter	88.9 ± 2.1^a^	63.9 ± 2.2^c^	82.1 ± 2.5^b^	75.3 ± 1.3^b^
	Caput lumen diameter	36.2 ± 1.3^a^	22.7 ± 0.7^b^	37.1 ± 1.1^a^	34.6 ± 1.0^a^
	Caput Epithelial height	24.3 ± 0.8^ab^	22.4 ± 0.5^b^	26.0 ± 0.9^a^	21.3 ± 0.6^b^
	Cauda diameter	121.8 ± 3.2^a^	111.1 ± 3.7^ab^	97.9 ± 2. 9^bc^	91.9 ± 2.7^c^
	Cauda lumen diameter	61.3 ± 2.7^ab^	42.4 ± 3.3^c^	51.4 ± 1.4^bc^	43.0 ± 3.0^c^
	Cauda Epithelial height	36.4 ± 1.2^a^	37.2 ± 1.2^a^	25.3 ± 1.0^c^	29.3 ± 0.9^b^
**Post-pubertal (56d)**	St. diameter	204.8 ± 4.0^c^	199.1 ± 4.2^c^	175.9 ± 3.1^d^	204.7 ± 5.2^c^
	St. Lumen diameter	64.4 ± 3.4^e^	84.2 ± 2.5^c^	86.7 ± 2.5^c^	75.2 ± 2.4^d^
	St. Epithelial height	70.2 ± 1.6^e^	56.7 ± 2.3^f^	49.0 ± 1.1^c^	60.2 ± 2.0^f^
	Caput diameter	128.7 ± 2.4^d^	115.3 ± 2.8^e^	121.2 ± 2.0^e^	131.4 ± 1.5^d^
	Caput lumen diameter	68.2 ± 1.4^d^	69.2 ± 1.7^d^	75.9 ± 1.3^c^	64.7 ± 1.7^d^
	Caput Epithelial height	27.9 ± 1.4^e^	28.1 ± 0.8^e^	40.4 ± 1.0^c^	32.8 ± 1.0^d^
	Cauda diameter	247.8 ± 6.4^d^	206.6 ± 6.4^e^	144.7 ± 3.4^g^	191.4 ± 4.6^f^
	Cauda lumen diameter	208.2 ± 8.2^d^	182.9 ± 5.7^e^	107.6 ± 3.7^g^	162.5 ± 4.2^f^
	Cauda Epithelial height	11.3 ± 0.8^e^	13.8 ± 0.9^e^	20.8 ± 1.0^d^	21.3 ± 1.2^d^

Values are expressed as mean ± SEM (n=6). Different labels indicate significant differences among groups within each column for different parameters at P<0.05. hyperthyroidism (Hyper), seminiferous tubule (St.).

The diameter of caput epididymis (CpE) was reduced significantly in all treated mice compared with controls at either times (24 d and 56 d), except for hyperthyroid animals during pubertal life. The lumen diameter of CpE was markedly decreased only in the D (39%) during short study period, although it was found significantly increased in the DH group (12%) compared with controls at the long study period. The epithelial height of CpE showed a tendency to increase in the DH group at both study periods, while it was found profoundly elevated (43%) in post-pubertal mice. The diameter of CdE was diminished by 42%, 23% and 17% in DH, H, and D groups, respectively, in adult mice; however, this decrement was noticed only in DH and H pre-pubertal animals compared to controls. We determined the lumen of CdE to be inversely proportional to the epithelial height of this segment, where the luminal diameter was found to be decreased significantly in DH (48%) and H (22%) groups compared with controls of adult mice; in contrast, their epithelium became wider. All these findings indicated some developmental impairments and inflammatory changes inside the tubes under the influence of diabetes and thyrotoxicosis. Collectively, hyperthyroidism induced through breastfeeding had minimal effects on the growth parameters of pre-pubertal mice; however, it might severely affect the histoarchitecture of these reproductive tubes when induced directly and concomitantly with diabetes.

### 3.4 Histopathology of experimental mouse testes

**Figure 3:**
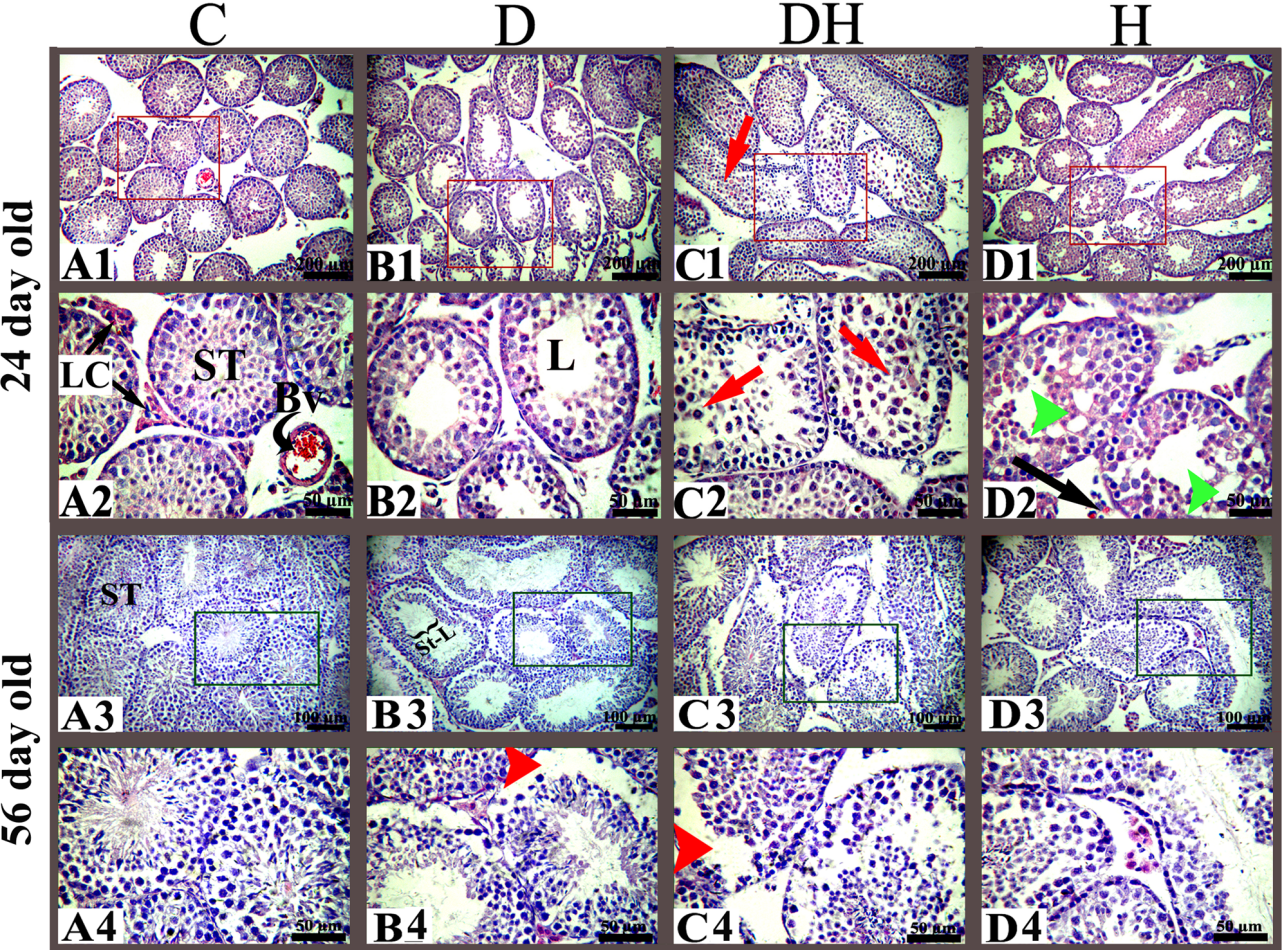
Histoarchitecture of the testes of pre- and post-pubertal mice exposed to diabetes and hyperthyroidism. control, C; diabetic, D; diabetic + hyperthyroidism, DH; hyperthyroidism, H; seminiferous tubule, ST; blood vessel, Bv; Leydig cell, LC; spermatogonia, SG; seminiferous tubule lumen, L. Representative images were captured at 100× and 400× magnifications. Different markings inside the images were inserted through Adobe Photoshop CS5, and the sizes of bars are 200 μm in the top row and 50, 100, and 50 μm in the subsequent bottom rows.

Histopathologic observations of testicular sections of pre-pubertal mice (**Figure 3**) revealed a well-organized germinal cellular lineage with normal size and number of cells inside the seminiferous tubules along with Leydig cells in the interstitial stroma. STZ-induced diabetic animals exhibited increased sizes of the lumen of seminiferous tubules, with a limited number of germinal cell and fewer underdeveloped cells (**Figure 3**; panels B1 and B2). We noted sloughed and undifferentiated germ cells in most of the tubes of the testes of the DH group animals (**Figure 3**, panels C1 and C2). We observed in the germinal epithelium of thyrotoxic subjects extensive vacuolization at many sites in the seminiferous tubules (**Figure 3**, panels D1 and D2).

**Figure 4:**
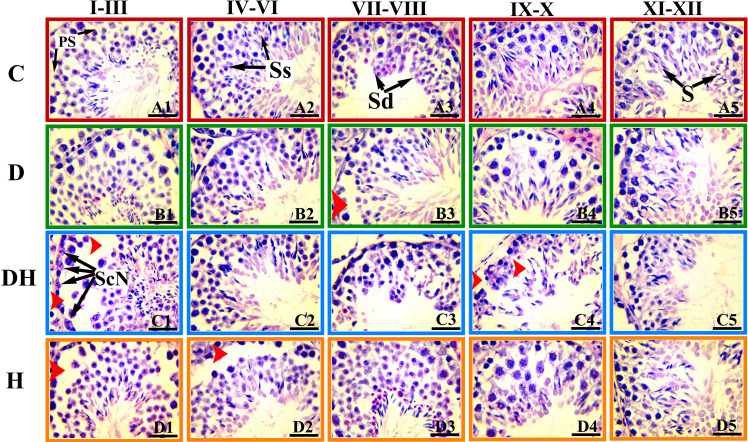
Photomicrographs of the testicular sections of adult mice treated with levothyroxine and STZ. These H&E-stained photographs were examined and captured under light microscopy at a magnification of 1000× usiing oil immersion. The images represent histopathologic changes at different stages of development of the seminiferous tubule at different stages of spermatogenesis. The stages are marked at the top of Figure and are denoted according to the staging method defined for the laboratory mouse (19). Ps, primary spermatocytes; Sc-N, Sertoli cell nuclei; Ss, secondary spermatocyte; Sd, spermatid; S, spermatozoa. Different markings within the images were inserted through Adobe Photoshop CS5, and bars are 30 μm in size.

Testicular sections of adult mice under different treatments exhibited a number of germ cells with fine structures in the control group (**Figures 3**, **4**). Many sites in the seminiferous tubules of D animals revealed missing stem cells and primary spermatocytes with increased luminal sizes (**Figure 3**, panels B3 and B4). Irregularly outlined seminiferous tubules with sloughed epithelial cells inside the lumen and disrupted cellular lineage were depicted in the DH group of mice (**Figure 3**, panels C3 and C4; **Figure 4**, panels C1–C5). However, hyperthyroid animals showed wider luminal diameters with a reduced number of cells near the basement membrane (**Figure 3**, panels D3 and D4; **Figure 4**, panels D1–D5).

### 3.5 Sperm count and motility measurements

**Figure 5:**
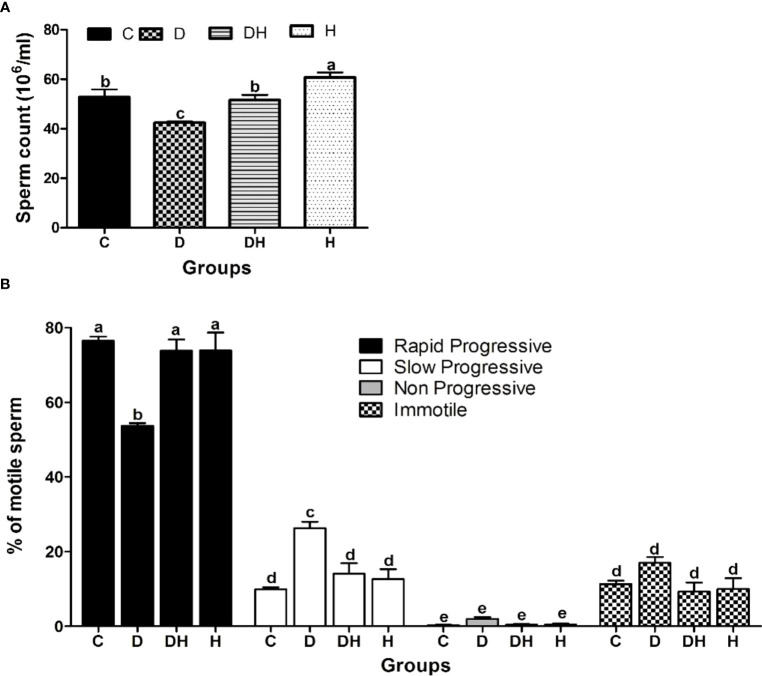
The effects of diabetes mellitus and hyperthyroidism on sperm concentration and percent motility in 8-week-old mice. **(A)** Each bar represents a mean ± SEM (n=12) and statistical differences were determined by 1-way ANOVA, followed by Tukey’s multiple-comparison test. Different labels indicate significant (p<0.05) differences among groups. The graph was created with GraphPad Prism, version 5.0. **(B)** Each bar represents a mean ± SEM (n=36), and different labels indicate significant (p<0.05) differences among groups. The graph was created with Graph-pad Prism, version 5.0.

Mice exposed to thyrotoxicosis and diabetes mellitus were assessed as to their fertility through sperm concentration and motility parameters (**Figure 5**). Sperm count was significantly reduced by 20% and increased 15% in D and H animals, respectively, compared with controls (**Figure 5A**). Rapid progressive sperm motility was significantly lower only in D (30%) compared with C mice (**Figure 5B**). Similarly, we noted a number of non-progressive sperm only in diabetic animals (**Figure 5B**).

## 4 Discussions

This is the first study where investigators have induced diabetes mellitus-plus-hyperthyroidism to ICR mice from neonatal to adult ages. We exposed the mice to these co-morbid metabolic diseases, and the fertility of the animals were scrutinized at both pre-pubertal (24 d) and post-pubertal (56 d of age) periods. Our results also clearly demonstrated that serum testosterone levels were increased by hyperthyroidism, but decreased by diabetes during peri-pubertal time. However, the interaction among testosterone, thyroid hormone and carbohydrate metabolism are so complex and require further studies.

We in the present study noted significant changes of body weights, testicular weights and epididymal weights, suggested that hyperthyroidism, induced concomitantly with diabetes, could exert adverse effects, while individual treatment with levothyroxine could have some beneficial effects.

Our study observed hair loss characters in over 80% of pre-pubertal mice in the DH and D groups, with either no body hair or relatively few hairs, respectively; however, this type of disorder disappeared when these animals entered puberty. Alopecia (hair loss) has proven to be the most prevalent of autoimmune diseases. Consistent findings of alopecia were reported in nonhuman primates manifesting various biologic dysfunctions, including endocrine disorders, immunologic diseases, and genetic mutations (20). Intriguingly, hair loss is one of the multiple cutaneous manifestations observed with endocrine disorders such as diabetes, and hyper- and hypothyroidism (19). These outcomes suggest that the rough skin coat or hair loss of treated mice during our experiment might be the result of a hormonal imbalance or some autoimmune reactions. Regardless, the detailed mechanisms involved still require elucidation.

The primary function of IGF is to control cellular production, differentiation, and apoptosis. However malfunctioning of the IGF axis has been shown to be linked to type-2 diabetes mellitus (T2DM) and pre-diabetic symptoms (21). It is believed that IGF-1 plays an important role in reducing the risk of T2DM. Peripherally, IGF-1 increases glucose uptake and functional inactivation of IGF1R in skeletal muscles of mice, resulting in insulin resistance and diabetes (22). Our present study found that hyperthyroidism markedly elevates IGF-1 and, conversely, STZ-induced diabetes suppresses IGF-1 from immaturity to adult age in mice, while in the DH syndrome group, thyrotoxic treatment caused up-regulation of IGF-1 to control levels. IGF-I and insulin have thus been proven to be crucial modulators of testicular functions and inducing a marked decrease in testicular IGF-I receptor content in STZ-diabetic rats (6). It is generally accepted that hyperthyroid animals show increased total testosterone levels in blood (23–25). Furthermore, the effects of thyroid hormones on male reproduction and fertility have been reviewed extensively; and it was concluded that despite increased total testosterone levels, thyrotoxic men experienced a relative androgen deficiency due to reduced free and bio-available testosterone and a relative rise in estrogen (26, 27). In accordance with reported data and our previous work (28), the present study showed elevated total testosterone values in hyperthyroid mice; however, the elevation was found to be distinctly diminished in diabetic subjects in both of our study periods. Decreased testosterone levels have also been demonstrated in STZ-diabetic animals (17, 29). Similarly, steroidogenesis is rouse promptly in Leydig cells by thyroid hormones. Thyroid hormones source multiplying of the cytoplasmic organelle peroxisome and excite the production of steroidogenic grave regulatory protein (StAR) and StAR mRNA in Leydig cells; both peroxisomes and StAR are linked with the passage of cholesterol, the essential in-between in steroid hormone biosynthesis, into mitochondria (5). The Leydig cells in the testes are responsible for testosterone production. Studies have shown that thyroid hormones regulate the function of Leydig cells by controlling the expression of enzymes involved in steroidogenesis, including cholesterol side-chain cleavage enzyme (CYP11A1) and 17α-hydroxylase (CYP17A1) (6). These findings indicate that reduced gonadotropins, either caused by diabetes or hyperthyroidism, were directly linked to male infertility.

The pre-pubertal mice in our study exhibited markedly reduced fT4 levels in the D group (21%) mice, and increased in the H group (31%), compared with controls. However, fT4 was diminished in D (16%) and elevated in H (22%) followed by DH (14%) animals compared with controls in the long-study group. Collectively, we observed the lowest values for fT4 in D animals at both times. During the short-study period, the levels of fT3 were significantly decreased in the D group (40%); however, these values were increased by 41% in the H group, compared with control mice. Similarly, in adult animals the values were much lower in D (44%) and higher in H groups (118%), compared with control subjects; although STZ-induced diabetes contravened the rise of fT3 values in the DH group in comparison to C animals at both times. These results suggest that induction of short-period hyperthyroidism in neonatal mice might be ameliorated by breastfeeding compared with treatments in adults.

The thyroid hormones (T3 and T4) are important key regulators of various biochemical actions, including lipid and carbohydrate metabolism, oxygen consumption, and several physiologic functions such as development, reproduction, and growth (30). The present study revealed diminished fT4 and fT3 levels in the serum of both pre- and post-pubertal STZ-diabetic mice; however, in contrast, these values were found profoundly elevated in levothyroxine (L-T4) treated animals. Consistently reduced levels of fT4 and fT3 have been reported in diabetic children, in spite of taking insulin (31). Current results are also in agreement with our previous work (14), where we observed higher concentrations of T3 and T4 in hyperthyroid immature rats. Research has shown that thyroid hormones can promote the proliferation and differentiation of spermatogonia, leading to the formation of mature sperm cells. In particular, studies have found that T3 can stimulate the expression of genes involved in spermatogonial differentiation and meiotic progression, leading to the formation of haploid sperm cells (7). However, excessive or insufficient levels of thyroid hormones can also have negative effects on spermatogonial development. For example, hypothyroidism (low thyroid hormone levels) has been associated with reduced sperm production and impaired spermatogonial development, while hyperthyroidism (excess thyroid hormone levels) can lead to increased oxidative stress and DNA damage in sperm cells (8).

Our observations revealed that the reduced size of seminiferous tubules along with undifferentiated germinal cells led to disturbed cellular lineage in hyperthyroid and concomitant diabetes plus hyperthyroid animals at 24 days of age. However, at short as well as long study periods, the sizes of the tubal lumen were elevated in D and DH group animals. Furthermore, the DH mice showed frequent residual bodies inside the seminiferous tubules at the short- (24 d), and sloughed germ cells during long-term (56 d) study. At the age of 24 d, only a few Leydig cells were extant in the testicular interstitium of DH and H groups compared with control mice. However, we saw many dispersed red blood cells near the basement membrane of seminiferous tubules in the testes of hyperthyroid animals at 8 weeks of age. The diverse spermatogenic cycles of seminiferous tubules were only observed in the testes of 56-d- old mice, whereas these cycles were rare at 24 d of age. The DH and H mice showed missing or dislocated primary spermatocytes and sloughed epithelial cells during observations of their cell cycle stages. During our study, reduced epithelial height and germ cell populations with abnormal cellular lineage were recorded in the seminiferous tubules of diabetic groups. Similar histoarchitecture of the testes with disruption of spermatogenesis in adult rats following a single intraperitoneal injection of STZ has also been reported previously (29). Furthermore, supportive evidences for the cellular abnormalities and irregular epithelial lining of seminiferous tubules with thyroid malfunction are seen in neonatal or prepubertal rodents (31). These findings demonstrate the importance of metabolic hormones for normal reproductive performance in male animals.

The basic morphologic and physiologic conditions of the epididymis are required for successful sperm transport together with regulation of their fertilizing capacity (32). The epididymis, being an important store house of spermatozoa, could be affected by direct and indirect disorders of the testes. We herein discovered intense adverse effects of STZ-diabetes–plus-hyperthyroidism on the epididymis of mice. We evaluated the proximal CpE and distal CdE of the ductus epididymis during the short (24 d) and long (56 d) study periods, and found that the normal control animals showed well organized, tall ciliated columnar cells (principal cells), basal cells, and clear cells; while concomitant diabetes mellitus-plus-hyperthyroidism critically damaged the ductulis efferentes and the ductus epididymis in DH subjects at both time periods. Others have also observed consistent histologic changes in pre-pubertal rats following STZ-induced diabetes (28, 33). The STZ-induced diabetic mice were severely affected, with reduced sperm count, progressive motility, and DNA integrity (34). A retrospective study (35) revealed a high prevalence of sub-fertility (51%) in diabetic patients, and consistently lower sperm concentration and motility were reported in diabetic rodents by other investigators (36–38). Similar to published studies, we found markedly reduced sperm count in DH and H (72%) groups followed by the D (12%) group, and progressive sperm motility was diminished by 81%, 42%, and 30% in DH, H, and D animals, respectively compared with control subjects. These findings suggest that diabetes and hyperthyroidism adversely affect the fertility of male animals, while concomitant polyglandular autoimmune disorders can exacerbate the effect toward infertility.

## Data availability statement

The raw data supporting the conclusions of this article will be made available by the authors, without undue reservation.

## Ethics statement

The animal study was reviewed and approved by the Institutional Animal Care and Use Committee of Nanjing Agricultural University, China.

## Author contributions

All authors contributed to the article and approved the submitted version.

## References

[1] Bhan G O'Brien T. Autoimmune endocrinopathy associated with diabetes insipidus. Postgraduate Med J (1982) 58:165. doi: 10.1136/pgmj.58.677.165 PMC2426350 7100039

[2] Al-Geffari M Ahmad NA Al-Sharqawi AH Youssef AM AlNaqeb D Al-Rubeaan K. Risk factors for thyroid dysfunction among type 2 diabetic patients in a highly diabetes mellitus prevalent society. Int J Endocrinol (2013) 2013:417920. doi: 10.1155/2013/417920 24454365 PMC3884781

[3] Eisenbarth GS Gottlieb PA. Autoimmune polyendocrine syndromes. New Engl J Med (2004) 350:2068–79. doi: 10.1056/NEJMra030158 15141045

[4] Ardestani SK Keshteli AH Khalili N Hashemipour M Barekatain R. Thyroid disorders in children and adolescents with type 1 diabetes mellitus in isfahan, Iran. Iranian J Pediatr (2011) 21:502. PMC3446149 23056839

[5] Association AD. Standards of medical care in diabetes—2013. Diabetes Care (2013) 36:S11. 23264422 10.2337/dc13-S011 PMC3537269

[6] Roubsanthisuk W Watanakejorn P Tunlakit M Sriussadaporn S. Hyperthyroidism induces glucose intolerance by lowering both insulin secretion and peripheral insulin sensitivity. J Med Assoc Thai (2006) 89:S133–40. doi: 10.2337/dc19-S008 17718254

[7] Donckier J. Endocrine diseases and diabetes. In: Pickup JC Williams G, editors. Text book of Diabetes mellitus. Chichester: Blackwell Publishing Company (2003). p. 27.21–5.

[8] Liu SC Wang Q Lienhard GE Keller SR. Insulin receptor substrate 3 is not essential for growth or glucose homeostasis. J Biol Chem (1999) 274:18093–9. doi: 10.1074/jbc.274.25.18093 10364263

[9] Radaideh A-RM Nusier MK Amari FL Bateiha AE El-Khateeb MS Naser AS. Thyroid dysfunction in patients with type 2 diabetes mellitus in Jordan. Saudi Med J (2004) 25:1046–50. 15322596

[10] Milionis C Ilias I Venaki E Koukkou E. Glucose homeostasis, diabetes mellitus, and gender-affirming treatment. Biomed (2023) 11(3):670. doi: 10.3390/biomedicines11030670 PMC10045127 36979649

[11] Victor SJ Horwitz EM Kini VR Martinez AA Pettinga JE Dmuchowski CF. Impact of clinical, pathologic, and treatment-related factors on outcome in patients with locally advanced breast cancer treated with multimodality therapy. Am J Clin Oncol (1999) 22:119–25. doi: 10.1097/00000421-199904000-00003 10199443

[12] KorejoOREJO NA Wei Q Zheng K Mao D Korejo RA Shah AH. Contemporaneous effects of diabetes mellitus and hypothyroidism on spermatogenesis and immunolocalization of Claudin-11 inside the seminiferous tubules of mice. BMC Dev Biol (2018) 18:15. doi: 10.1186/s12861-018-0174-4 29940839 PMC6019809

[13] Ariza L Pagès G García-Lareu B Cobianchi S Otaegui P Ruberte J. Experimental diabetes in neonatal mice induces early peripheral sensorimotor neuropathy. Neuroscience (2014) 274:250–9. doi: 10.1016/j.neuroscience.2014.05.015 24846610

[14] Vergouwen R Huiskamp R Bas R Roepers-Gajadien H Davids J De Rooij D. Postnatal development of testicular cell populations in mice. J Reprod fertil (1993) 99:479–85. doi: 10.1530/jrf.0.0990479 8107030

[15] Song Y-H Li Y Maclaren NK. The nature of autoantigens targeted in autoimmune endocrine diseases. Immunol Today (1996) 17:232–8. doi: 10.1016/0167-5699(96)10008-6 8991385

[16] Gong T Wei Q-W Mao D-G Nagaoka K Watanabe G Taya K. Effects of daily exposure to saccharin and sucrose on testicular biologic functions in mice 1. Biol Reprod (2016) 95:116, 111–113. doi: 10.1095/biolreprod.116.140889 27683267

[17] Wennemuth G Westenbroek RE Xu T Hille B Babcock DF. CaV2. 2 and CaV2. 3 (N-and R-type) Ca2+ channels in depolarization-evoked entry of Ca2+ into mouse sperm. J Biol Chem (2000) 275:21210–7. doi: 10.1074/jbc.M002068200 10791962

[18] Afriani T Jaswandi, Rastosari A Al Razzak MC Wahyudi D. Addition of tomato juice as additive in diluent of egg yolk citrate on the quality of Pesisir Cattle Semen. J Anim Health Prod (2023) 11(1):62–7. doi: 10.17582/journal.jahp/2023/11.1.62.67

[19] Jabbour SA. Cutaneous manifestations of endocrine disorders. Am J Clin Dermatol (2003) 4:315–31. doi: 10.2165/00128071-200304050-00003 12688837

[20] Novak MA Meyer JS. Alopecia: possible causes and treatments, particularly in captive nonhuman primates. Comp Med (2009) 59:18–26. 19295051 PMC2703143

[21] Sandhu M. Insulin-like growth factor-I and risk of type 2 diabetes and coronary heart disease: molecular epidemiology. In: IGF-I and IGF Binding Proteins. Basel (Switzerland): Karger Publishers (2005) 9:44–54. 10.1159/000085755 15879687

[22] Fernández AM Kim JK Yakar S Dupont J Hernandez-Sanchez C Castle AL. Functional inactivation of the IGF-I and insulin receptors in skeletal muscle causes type 2 diabetes. Genes Dev (2001) 15:1926–34. doi: 10.1101/gad.908001 PMC312754 11485987

[23] Aruldhas MM Valivullah HM Srinivasan N Govindarajulu P. Role of thyroid on testicular lipids in prepubertal, pubertal and adult rats. I. Hyperthyroidism. Biochim Biophys Acta (BBA)-General Subj (1986) 881:462–9. doi: 10.1016/0304-4165(86)90040-1 3697378

[24] Zähringer S Tomova A Von Werder K Brabant G Kumanov P Schopohl J. The influence of hyperthyroidism on the hypothalamic-pituitary-gonadal axis. Exp Clin Endocrinol Diabetes (2000) 108:282–9. doi: 10.1055/s-2000-7756 10961359

[25] Ford H Cooke R Kelghtley E Feek C. Serum levels of free and bound testosterone in hyperthyroidism. Clin Endocrinol (1992) 36:187–92. doi: 10.1111/j.1365-2265.1992.tb00956.x 1568351

[26] Krajewska-Kulak E Sengupta P. Thyroid function in male infertility. Front Endocrinol (2013) 4:174. doi: 10.3389/fendo.2013.00174 PMC3826086 24312078

[27] Aschebrook-Kilfoy B Sabra MM Brenner A Moore SC Ron E Schatzkin A. Diabetes and thyroid cancer risk in the National Institutes of Health-AARP Diet and Health Study. Thyroid (2011) 21:957–63. doi: 10.1089/thy.2010.0396 PMC3162644 21767143

[28] Korejo NA Wei Q-w Shah AH Shi F. Effects of concomitant diabetes mellitus and hyperthyroidism on testicular and epididymal histoarchitecture and steroidogenesis in male animals. J Zhejiang University-SCIENCE B (2016) 17:850–63. doi: 10.1631/jzus.B1600136 PMC5120227 27819132

[29] Deeds M Anderson J Armstrong A Gastineau D Hiddinga H Jahangir A. Single dose streptozotocin-induced diabetes: considerations for study design in islet transplantation models. Lab Anim (2011) 45:131–40. doi: 10.1258/la.2010.010090 PMC3917305 21478271

[30] Kundu S Pramanik M Roy S De J Biswas A Ray AK. Maintenance of brain thyroid hormone level during peripheral hypothyroid condition in adult rat. Life Sci (2006) 79:1450–5. doi: 10.1016/j.lfs.2006.04.006 16698041

[31] Artimani T Amiri I Asl SS Saidijam M Hasanvand D Afshar S. Amelioration of diabetes‐induced testicular and sperm damage in rats by cerium oxide nanoparticle treatment. Andrologia (2018) 50(9):e13089. doi: 10.1111/and.13089 30022501

[32] James ER Carrell DT Aston KI Jenkins TG Yeste M Salas-Huetos A. The role of the epididymis and the contribution of epididymosomes to mammalian reproduction. Int J Mol Sci (2020) 21(15):5377. doi: 10.3390/ijms21155377 32751076 PMC7432785

[33] Radetti G Paganini C Gentili L Barbin F Pasquino B Zachmann M. Altered adrenal and thyroid function in children with insulin-dependent diabetes mellitus. Acta Diabetologica (1994) 31:138–40. doi: 10.1007/BF00570367 7827351

[34] Mangoli E Talebi AR Anvari M Pourentezari M. Effects of experimentally-induced diabetes on sperm parameters and chromatin quality in mice. Iranian J Reprod Med (2013) 11:53. PMC3941382 24639693

[35] La Vignera S Calogero A Condorelli R Lanzafame F Giammusso B Vicari E. Andrological characterization of the patient with diabetes mellitus. Minerva endocrinologica (2009) 34:1–9. 19209124

[36] Scarano W Messias A Oliva S Klinefelter G Kempinas W. Sexual behaviour, sperm quantity and quality after short-term streptozotocin-induced hyperglycaemia in rats. Int J androl (2006) 29:482–8. doi: 10.1111/j.1365-2605.2006.00682.x 16524366

[37] Kim ST Moley KH. Paternal effect on embryo quality in diabetic mice is related to poor sperm quality and associated with decreased glucose transporter expression. Reproduction (2008) 136:313–22. doi: 10.1530/REP-08-0167 18558660

[38] Xu R Wang F Zhang Z Zhang Y Tang Y Bi J. Diabetes-induced autophagy dysregulation engenders testicular impairment via oxidative stress. Oxid Med Cell Longev (2023) 2023:4365895. doi: 10.1155/2023/4365895 36778206 PMC9918358

